# Antibacterial Properties of Rose Bengal Conjugated to Hyaluronic Acid

**DOI:** 10.3390/ijms25063330

**Published:** 2024-03-15

**Authors:** Melad Atrash, Iryna Hovor, Yanna Gurianov, Margarita Barel, Olga Semenova, Tamara Brider, Marina Nisnevitch, Faina Nakonechny

**Affiliations:** Department of Chemical Engineering, Ariel University, Kiryat-ha-Mada, Ariel 4070000, Israel

**Keywords:** rose bengal, hyaluronic acid, conjugation, *S. aureus*, *E. coli*, photodynamic antibacterial activity, sonodynamic antibacterial activity, slow drug release

## Abstract

Dental diseases, including conditions affecting oral structures, have become more common due to unhealthy lifestyle choices. Traditional antibiotic treatments face challenges related to the development of antibiotic resistance in bacteria. Photodynamic antibacterial chemotherapy is emerging as a promising alternative using photosensitizers to generate reactive oxygen species upon exposure to light. This article examines the photosensitizer Rose Bengal (RB) immobilized in hyaluronic acid (HA) for prolonged antibacterial action. The RB-HA conjugate demonstrated a molar ratio of approximately three RB residues to each of the ten units of HA. RB-HA exhibited a high singlet oxygen quantum yield (ΔΦ = 0.90), suggesting its efficacy in photodynamic treatment. A photostability analysis revealed slower photobleaching of RB-HA, which is essential for prolonged application. Under visible light and ultrasonic treatment, RB-HA exhibited effective antibacterial activity against Gram-positive *S. aureus* and Gram-negative *E. coli* bacteria for at least 80 days. The gradual release of RB ensured sustained bactericidal concentration. The study establishes RB-HA as a promising candidate for antimicrobial photodynamic and sonodynamic therapy in dental and other medical fields, providing enhanced stability and prolonged antibacterial efficacy.

## 1. Introduction

Dental diseases, also known as oral diseases, encompass conditions that affect the teeth, gums, and other oral structures. Unhealthy lifestyle choices, such as unhealthy diets, processed foods, poor oral hygiene, and chronic stress, have contributed to the increased prevalence of these diseases [1,2,3,4]. As a consequence of these lifestyle choices, a significant portion of the population suffers from different dental problems, including periodontal diseases (gum diseases), and oral infections [5]. Without antibacterial treatment, these may cause damage to the supporting system of the tooth, including gum recession and bone loss in the jaw [6,7]. In many cases, the treatment of these bacteria is accomplished using antibiotics. However, the growth and spread of antibiotic-resistant bacteria can pose problems in the treatment of oral and dental diseases. Therefore, there is a need to search for new approaches to mitigate pathogenic bacteria [8,9,10].

Photodynamic Antibacterial Chemotherapy (PACT) presents promising prospects as an alternative to antibiotic treatment [11]. This therapy is performed using photosensitizers (PSs), which generate reactive oxygen species (ROS) under light [12]. This antimicrobial approach has a multi-targeted nature, making resistance development by bacteria unlikely. ROS in microbial cells damage various targets non-specifically, including nucleic acids, membrane-bound protein complexes, cytosolic enzymes, plasma membranes, and lipids. It is also supposed that PSs do not need to enter microbial cells, as adhesion to cell walls and membranes is enough for bacterial killing, bypassing resistance development [13]. PACT has become an essential tool in the field of dentistry, being used in a wide range of specialized areas such as general dentistry, periodontics, orthodontics, endodontics, surgery, and implantology [14,15,16]. An additional method of the activation of PSs in the treatment of diseases of the oral cavity can be ultrasound. By combining ultrasound with photosensitizing agents, it is possible to enhance therapeutic outcomes [17]. Ultrasound can efficiently penetrate oral tissues, reaching areas that are difficult to access using conventional light sources. Ultrasound also activates PSs, leading to the production of ROS, which are responsible for the destruction of targeted oral pathogens. The use of PSs has advantages, such as reducing overall treatment time, eliminating the need for patient sedation, destroying bacterial cells, and combating multiresistant pathogens [18]. Additionally, PSs can be used in conjunction with other dental procedures, providing a non-invasive and versatile treatment option for a wide range of oral diseases [19]. However, saliva can dilute active ingredients in the mouth, biofilms reduce drug effects on microorganisms, and delivering drugs to specific sites such as the periodontal pocket is challenging. Therefore, carrier systems are required to consistently release them for effective concentrations. Advanced biomaterials like hydrogels, films, and nanoparticles hold promise as carriers for local drug delivery. These delivery systems offer advantages such as increasing drug solubility, prolonging action time, and reducing cytotoxicity [20,21].

Rose Bengal (RB) is a well-known PS that has strong antibacterial effects, which are not limited to a specific bacterial strain [22,23,24]. The probable bactericidal mechanism of RB involves the penetration of RB through bacterial cell walls and its binding to cell membranes, followed by the generation of reactive oxygen species under illumination conditions [25]. The high photodynamic activity of RB has been demonstrated against a variety of Gram-positive and Gram-negative bacteria, as well as against biofilms [25,26,27]. However, for medical use, it should be used in combination with materials that inhibit its rapid elimination from the body, allow it to concentrate exclusively in areas of inflammation, and exhibit a repeated effect.

The literature describes various approaches in which RB was combined with both organic and inorganic materials. In our previous works, we reported on RB immobilized in polymers to enable their reusability or continuous use. For instance, RB immobilized in low-density polyethylene through co-extrusion kept its photodynamic antibacterial activity for at least five days, and in polypropylene—for 11 days [28]. RB immobilized in polystyrene films effectively destroyed *S. aureus* and *E. coli* under illumination by white light for a week [29], and wastewater bacteria—for a month [30]. In addition, organically modified silica matrices with incorporated RB illuminated with white light kept high activity against bacteria for more than 10 cycles of use [31].

RB also exhibited photodynamic antibacterial properties when bound to a tissue–mimetic collagen matrix [32], chitosan [33], and furfuryl-modified gelatin [34]. However, in these studies, the reusability or continuous use of RB has not been examined.

Based on the literature data, we chose hyaluronic acid (HA) as a matrix for conjugation with RB and obtaining a long-term PS for the treatment of oral infections. HA has multiple medical applications due to high hygroscopicity and good biocompatibility when the half-life of HA in humans is weeks [35]. We assumed that the conjugation of RB with an HA matrix would possibly gradually release RB over an extended period, maintaining an RB concentration sufficient for antibacterial activity specifically in the target area, thus minimizing potential side effects.

The aim of the present work was to study the in vitro effects of the conjugate RB-HA against Gram-positive (*S. aureus*) and Gram-negative (*E. coli*) bacteria under white light illumination and ultrasonic treatment.

## 2. Results and Discussion

### 2.1. Characterization of RB-HA Conjugate

#### 2.1.1. Composition of RB-HA Conjugate

To determine the composition of the conjugate, the latter was dissolved in NaOH solution, and the RB concentration was measured using a calibration curve of free RB dissolved in NaOH of the same concentration. Then, the content of RB in the conjugate and the molar ratio of RB to HA in the conjugate was calculated, which was 0.31, i.e., approximately three RB residues are bonded to each ten units of HA.

#### 2.1.2. FTIR Assay of RB-HA

**Figure 1:**
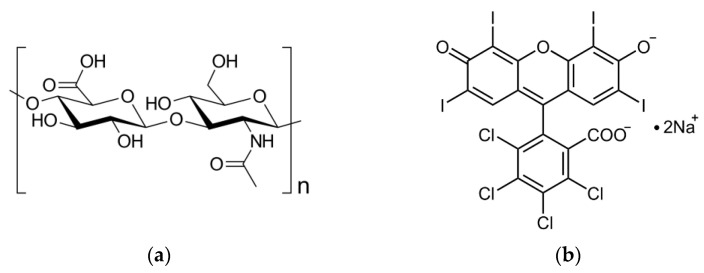
The structural formulas of hyaluronic acid (HA) (**a**) and Rose Bengal (RB) (**b**).

**Figure 2:**
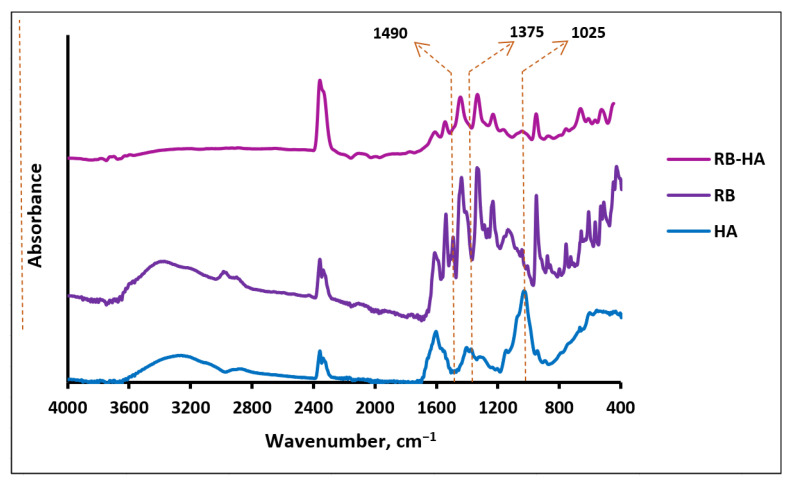
The FTIR spectra of HA, RB, and RB-HA.

The HA structure includes carboxyl and hydroxyl functional groups and ether and amide bonds (Figure 1a), which are reflected in the FTIR spectrum of HA (Figure 2) with bands at 3282 cm^−1^ (a broad band of OH and NH stretching), 2859 cm^−1^ (NH stretching), 1601 cm^−1^ (NH bending) [36], 1539 cm^−1^ and 1402 cm^−1^ (antisymmetric and symmetric stretching of the amide group, respectively) [37], 1375 cm^−1^ and 1355 cm^−1^ (OH bending in carboxyl and hydroxyl groups, respectively), 1126 cm^−1^ (ester CO stretching) [36], and 1025 cm^−1^ (the vibrational modes of -CH_2_OH groups of carbohydrates) [38]. The FTIR spectrum of RB contains bands corresponding to its structure (Figure 1b and Figure 2): 3360 cm^−1^ and 2969 cm^−1^ (OH stretching) [36], 1609 cm^−1^ (CH bending in the aromatic ring) [36], 1232 cm^−1^ and 1127 cm^−1^ (CO stretching) [36], 1439 cm^−1^ (aromatic ring C–C stretching) [39], 1412 cm^−1^ and 950 cm^−1^ (OH bending) [36], 757 cm^−1^, 657 cm^−1^ and 610 cm^−1^ (C–Cl stretching), and 566 cm^−1^ and 516 cm^−1^ (C–I stretching) [36]. Two bands, 1540 and 1490 cm^−1^, are assigned to asymmetrical and symmetrical stretching vibrations of COO^–^, respectively [40]. Actually, carboxylates are characterized by two intense bands in the ranges of 1650–1540 cm^−1^ and 1450–1360 cm^−1^, and the 1540 cm^−1^ signal in the RB spectrum can be definitely related to the carboxylate group, but although the 1490 cm^−1^ band seems to lie outside the 1450–1360 cm^−1^ range, we still assign it to COO^–^, explaining this shift through dipole–dipole interactions [41,42]. In the spectrum of the RB-HA conjugate (Figure 2), the bands at 1375 cm^−1^ and 1490 cm^−1^ assigned to OH bending in carboxyl groups and symmetrical stretching vibrations of COO^–^, respectively, are absent. These changes prove the involvement of these groups in the formation of new bonds in the RB-HA conjugate. In addition, a band at 1025 cm^−1^, typical for the -CH_2_OH groups of carbohydrates and is thus absent in the spectrum of RB, appears in the spectrum of the conjugate along with bands characteristic for RB groups. Since RB and HA are connected through diaminopropane, providing amide bonds, which are already present in the HA structure (Figure 1a), the appearances of new amide bonds cannot be registered in the FTIR spectrum of the conjugate. The bands 2359 cm^−1^ and 2334 cm^−1^, which are present in all the spectra, are due to characteristic vibrations of CO_2_ in the air [36].

### 2.2. Generation ROS by RB-HA

**Figure 3:**
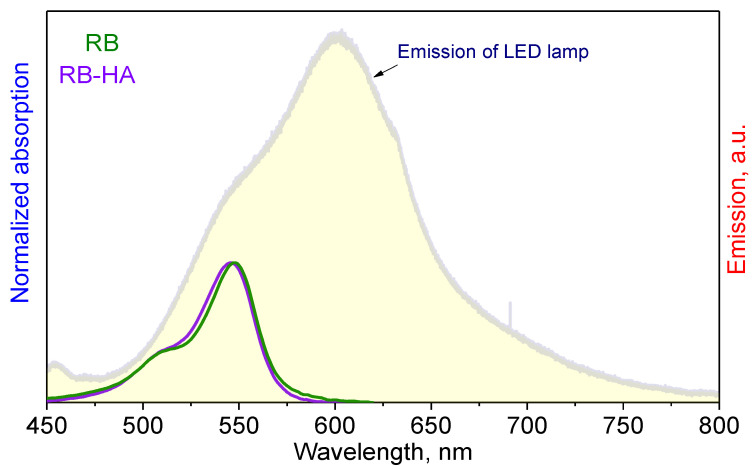
The spectral overlap between the absorption spectra of the RB and RB-HA in saline and the emission spectrum of the LED lamp.

The formation of ROS is the most significant property of PSs. The singlet oxygen quantum yield (ΔΦ), or the amount of singlet oxygen produced, characterizes the abilities of molecules to be PSs in PDT applications [18]. It is known that RB generates free oxygen radicals when exposed to light [22,43]. However, it was necessary to test whether the RB-HA conjugate was capable of producing ROS as well. Absorption spectra of RB and RB-HA in saline were measured and compared to the emission spectrum of the white LED lamp used in this study (Figure 3). It can be seen that the absorption spectra of the compounds fully overlap with the emission spectrum of the LED lamp, and therefore, this light source can be applied in photodynamic studies.

**Figure 4:**
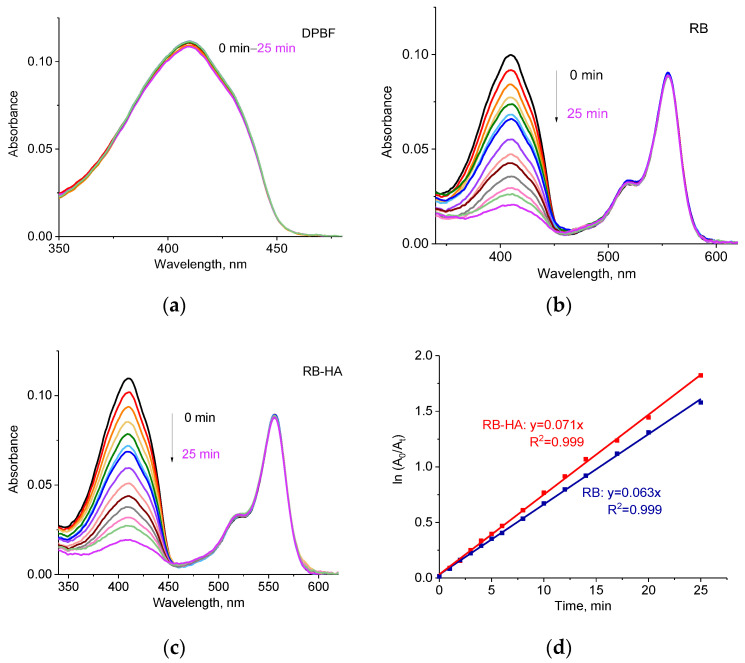
(**a**) Absorption spectra of DPBF under LED lamp irradiation; absorption spectra of RB (**b**) and RB-HA (**c**) with DPBF under LED lamp irradiation; time-dependent decomposition of DPBF through singlet oxygen produced by RB and RB-HA (**d**).

The singlet oxygen generation by RB-HA under the LED lamp was measured in methanol using DPBF and compared to the ROS generation by RB (Figure 4). DPBF is a fluorescent probe that reacts with some ROS, resulting in the formation of endoperoxides, which leads to the decomposition of the probe [44,45,46]. This process can be monitored spectrophotometrically since this process is accompanied by a decrease in the absorption of DPBF at 410 nm. Figure 4a shows that the absorption of DPBF alone did not change in time. However, the absorption of DPBF drops drastically when irradiated in the presence of RB and RB-HA (Figure 4b,c). The decrease in the absorption in time enabled us to calculate the DPBF degradation rate constant *k* (Figure 4d) [47,48]. The degradation rate constants for RB and RB-HA were 0.063 min^−1^ and 0.071 min^−1^, respectively. The difference between these values was not statistically significant (*p*-value = 0.68), suggesting that the ROS generation by RB is not substantially altered when it is conjugated.

The obtained constants allowed for the determination of the singlet oxygen quantum yield (ΔΦ) of RB-HA, using the following equation [49,50]:

ΔΦ_RB-HA_ = ΔΦ_RB_ × *k*_RB-HA_/*k*_RB_

The singlet oxygen quantum yield of RB was reported to be ΔΦ = 0.80 in methanol [51], and that calculated for RB-HA was ΔΦ = 0.90. Thus, the conjugate demonstrated a higher rate of singlet oxygen production than the free RB, which shows the potential of this compound as a PS in PDT applications.

Interestingly, ROS formation was not observed under ultrasonic treatment. The behavior of DPBF in the presence of RB or RB-HA did not differ from that of DPBF alone.

### 2.3. Photostability of RB-HA Conjugate

The important factor in PDT is the photostability of the PS since a high rate of photodegradation under illumination may lead to insufficient ROS production and make the PS not applicable for effective PDT. The photostability of PSs can be monitored through changes in the absorption spectra, during light irradiation.

RB is known to be photostable since its half-life time, or the time it takes the concentration of a reactant to reach half of its initial value, reaches values of tens of minutes [52,53]. The photostability of the RB-HA conjugate was compared to that of free RB and tested under the experimental conditions used for illumination in photodynamic experiments. The initial optical densities of RB and RB-HA were equal.

**Figure 5:**
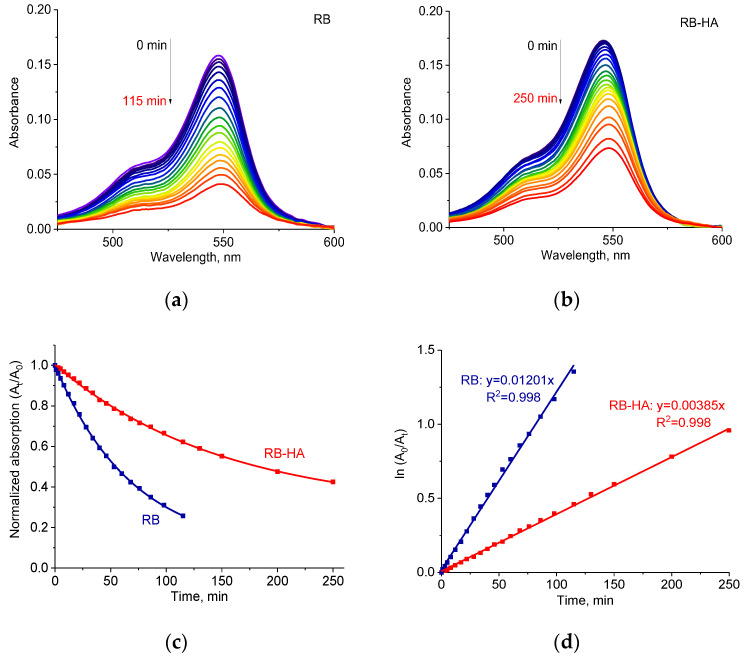
Absorption spectra of RB (**a**) and RB-HA (**b**) under light irradiation by LED lamp and time-dependent decomposition of RB and RB-HA (**c**,**d**). A_0_ and A_t_ are absorption values at zero and illumination time periods.

Figure 5 shows decreases in the absorption spectra of RB (a) and RB-HA (b) under irradiation by LED lamp. The drop in normalized absorbance at 547 nm in time was less considerable in the case of conjugated RB than for the free one (Figure 5c). The absorption decay half-lives were calculated from the obtained monoexponential decay functions and were 48 min and 104 min for RB and RB-HA, respectively. The calculated kinetic constants of decomposition rate (*k*), determined as a slope of the ln(A_0_/A_t_) vs. time plot (Figure 5d) [54], were *k* = 0.01201 min^−1^ for RB and *k* = 0.00385 min^−1^ for RB-HA, which indicated that the process of the photodegradation of RB-HA was three times slower than of RB. This difference was proven statistically with a *p*-value of 0.0014. We consider that RB-HA exhibits higher photostability than RB, which is crucial for PDT applications.

### 2.4. Photo- and Sonodynamic Activity of RB-HA

**Figure 6:**
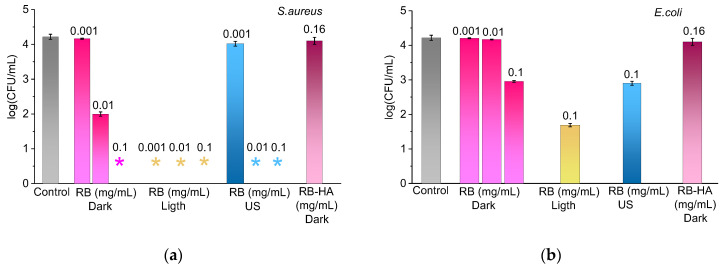
The effects of RB and RB-HA at different concentrations in the inactivation of *S. aureus* (**a**) and *E. coli* (**b**) cells at concentrations of 10^4^ CFU/mL in the dark, under illumination and ultrasonic treatment. The asterisk * indicates the total inhibition of bacterial cells.

First of all, the dark toxicity of RB-HA was examined, as high concentrations of free RB are known to reduce cell viability without irradiation, a phenomenon referred to as dark antibacterial toxicity [55,56]. In this case, the inhibition of bacterial growth occurs in a manner distinct from the photodynamic mechanism, when the photoexitation of PSs involves the excitation of triplet oxygen, leading to the production of cytotoxic reactive oxygen species. Actually, the mechanism of dark toxicity is still being investigated [25]. To study the dark antibacterial activity of RB-HA, cells of *S. aureus* and *E. coli* were incubated in the dark in the presence of free or conjugated RB at various concentrations, and then, the cell viability was tested (Figure 6). At the same time, the antibacterial activity of free RB was tested under light and ultrasound irradiation (Figure 6).

The experiment showed that in the dark, *S. aureus* was eradicated at an RB concentration of 0.1 mg/mL, whereas at 0.01 mg/mL, the cells were only partially inactivated, and at 0.001 mg/mL, remained intact (Figure 6a). Moreover, at all three RB concentrations, *S. aureus* was eradicated under exposure to white light. When treated using ultrasound, bacteria survived only at the lowest RB concentration of 0.001 mg/mL. Another situation was seen in the case of *E. coli*, when, only at 0.1 mg/mL RB, the cell concentration decreased by 1 log_10_ (Figure 6b), and RB at 0.001 and 0.01 mg/mL did not affect the viability of *E. coli*. Under illumination and ultrasonic irradiation in the presence of 0.1 mg/mL RB, the count of alive cells was lower than at dark conditions, but the complete eradication of *E. coli* cells was not reached. The results obtained for both bacteria were compared to a slightly higher concentration of RB-HA 0.16 mg/mL. As can be seen in Figure 6, at this concentration of the conjugate, the bacteria completely survived. Thus, this concentration was used for subsequent experiments.

The bacterial survival was also examined under LED light and sonication in the absence of RB and RB-HA. Cells of *S. aureus* treated for 10 min, and of *E. coli,* for 20 min, remained intact and served as a control in each case. The treatments during 10 min for S. aureus and 20 min for *E. coli* had no impact on viability.

To estimate the antibacterial activity of RB-HA, equal portions of the conjugate were distributed into a number of glass transparent vials. Once or twice a week, cells of Gram-positive S. aureus and Gram-negative E. coli bacteria were added to the vials, either illuminated or sonicated, and then tested for viability.

**Figure 7:**
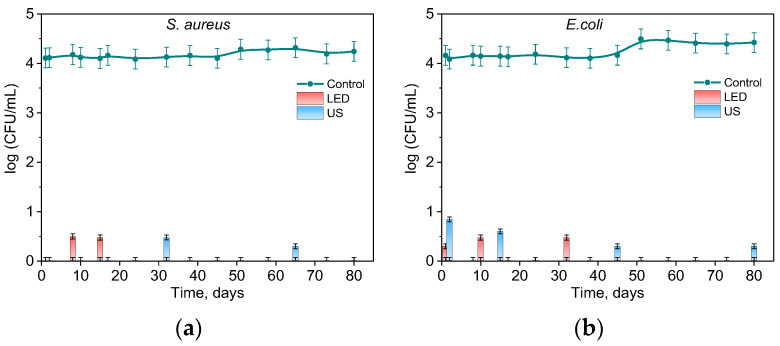
Inhibition of *S. aureus* (**a**) and *E. coli* (**b**) by RB-HA under illumination (LED) and sonication (US). Control — cells, illuminated or sonicated in the absence of RB-HA.

Figure 7 demonstrates the antibacterial activity of the RB-HA conjugate against *S. aureus* (Figure 7a) and *E. coli* (Figure 7b) upon illumination with visible light and ultrasonic treatment. It can be seen that RB-HA was effective in killing both types of microorganisms and kept its activity under repeated use for at least 80 days. In all cases, the bacteria were either totally killed or strongly inhibited when their concentration dropped by ca. 3.5 log_10_.

**Figure 8:**
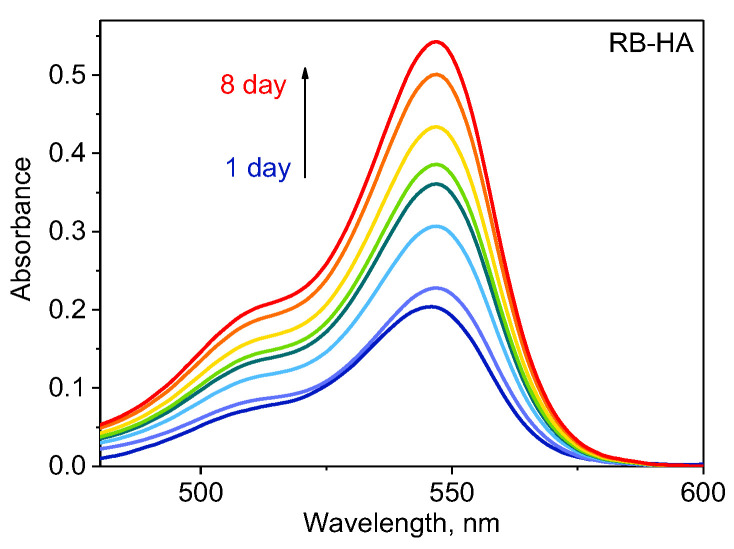
UV spectra of RB-HA in saline kept in the dark at an ambient temperature.

We assumed that the continuous gradual release of RB from the conjugate provided a long-term bactericidal concentration of PS in the medium. To examine this possibility, the solution of RB-HA in saline was incubated for 8 days in the dark and monitored using a measurement of absorbance (Figure 8).

As can be seen from Figure 8, during the experiment, the absorption increased in time. The obtained result demonstrated a gradual release of RB from the conjugate. This observation is also confirmed by changes in the RB concentration in the solutions used in the experiments for testing antibacterial activity. In the experiment on testing the antibacterial activity of RB-HA, on day 80, the concentration of free RB in the vials that underwent illumination was 0.018 mg/mL, and in those that were treated with ultrasound — 0.23 mg/mL. Thus, the long-term antibacterial effect of RB-HA under illumination is explained by the high ability of the conjugate to produce a high rate of ROS when the concentration of released RB was definitely not sufficient for the dark activity of RB. In the case of ultrasound treatment, we showed that RB did not generate ROS under our experimental conditions. However, the sonication of the conjugate led to a significant increase in the concentration of free RB up to that of dark toxicity. In addition, ultrasonic treatment is known to improve cell sensitivity to RB [29,57]. These factors led to the complete killing of bacteria.

## 3. Methods and Materials 

### 3.1. Synthesis and Characterization of RB-HA Conjugate

All the chemicals used in this study were of analytical grade: hyaluronic acid (HA)—MW 30–50 kDA, Glentham Life Sciencs Ltd., Corsham, England; 1,2-Diaminopropane, 99% (PDA)—Thermo Fisher Scientific, Fair Lawn, NJ, USA; carbodiimide [N-ethyl-N = -(3-dimethylaminopropyl) carbodiimide (EDC)—Glentham Life Sciencs Ltd., Corsham, England; hydroxybenzotriazole (HOBt)—Sigma-Aldrich Chemie GmbH, Steinheim, Germany; and Rose Bengal (RB)—Alfa Aesar, Heysham, England.

The conjugate RB-HA was synthesized using chemical cross-linking via EDC using a PDA linker. The solution of HA (0.1 g) was prepared in 30 mL of distilled water and stirred in a shaker until the HA dissolved. Then, 0.4 mL of PDA was added to the HA solution and stirred using the vortex. EDC (1.15 g, 6 mM) and HOBt (0.81 g, 6 mM) were dissolved in a 20 mL mixture of DMSO–distilled water (1:1). The obtained solutions were mixed in a flask and stirred overnight at 120 rpm. After that, 20 mL of a solution containing RB (1 g, 51.4 mM), EDC (1.15 g, 6 mM), and HOBt (0.81 g, 6 mM) in a mixture of DMSO–distilled water (1:1) were added, and the solution was further stirred in the dark. The conjugated product was then dialyzed against distilled water using a dialysis membrane (cellulose tubing; Sigma-Merck, Darmstadt, Germany) for one week and then lyophilized. The obtained conjugate RB-HA was stored at −20 °C in dark tubes.

### 3.2. Checking the RB Load in the Conjugate

To assess the RB load in the conjugate, an accurately weighed amount of 10 mg of the conjugate was dissolved in 10 mL of 1 M NaOH solution, and its absorbance was measured at 548 nm using a spectrophotometer (Evolution 201; Thermo Fisher Scientific Inc., Madison, WI, USA), and the RB concentration was calculated using a calibration curve. It enabled us to calculate the amount of the released RB and to calculate the mass and molar ratio between the RB and HA.

### 3.3. FTIR Analysis

The samples were analyzed using an FTIR spectrometer (Jasco, 6800 FV, Tokyo, Japan) at room temperature in the 4000–400 cm^−1^ range at an operation number of 64 scans, a resolution of 2.0 cm^−1^, and a scanning interval of 1 cm^−1^. The spectra were analyzed using Spectra Analysis™ (Jasco, Tokyo, Japan).

### 3.4. Spectral Studies and Photostability Tests

The emission spectrum of the white LED lamp used for illumination was registered using the HORIBA spectrometer (Jobin Yvon Inc., Edison, NJ, USA) equipped with a monochromator FHR1000 and detector CCD (HORIBA Scientific’s Synapse™, Irvine, CA, USA).

Photostability was measured in saline in 1 cm quartz cuvettes at RT during irradiation with the LED lamp. Absorption spectra were recorded before light treatment and during irradiation using a Thermo Scientific Evolution 201 spectrophotometer. The initial absorbance values of the RB and RB-HA at the absorption maximum were ~0.16. The ratios (A_t_/A_0_) and ln (A_0_/A_t_) of the measured absorbencies at the long-wavelength maximum before (A_0_) and after exposure (A_t_) were used to plot decay curves. The photostability experiment for each sample was performed 3 times and the average decay curve was obtained.

### 3.5. Generation of ROS

The singlet oxygen generation (ROS) of RB-HA was measured in methanol with 1,3-diphenylisobenzofuran (DPBF) as the singlet oxygen scavenger, following the procedure in [58]. RB was used as the reference dye. The initial absorbance values of the RB and RB-HA at 555 nm were ~0.09. Samples were light-irradiated in a standard 1 cm quartz cuvette using the LED lamp. The corresponding plot ln(A_0_/A_t_) vs. time, representing the absorbance of DPBF at 410 nm at zero (A_0_) and during irradiation (A_t_), was drawn and fitted using a first-order reaction rate function.

### 3.6. Bacterial Growth

Cultures of Gram-positive bacteria *S. aureus* (ATCC 25923) and Gram-negative bacteria *E. coli* (ATCC 25922) were grown on Brain Heart agar plates (BHA, Acumedia, Lansing, MI, USA) for 24 h, transferred into Brain Heart broth (BH, Acumedia, Lansing, MI, USA), grown at 37 ± 1 °C with shaking at 170 rpm until reaching the absorbance of OD_660_ ≈ 0.3, and diluted with commercially available sterile 0.9% saline solution to OD_660_ = 0.10 ± 0.02, which corresponds to a cell concentration of 10^8^ CFU mL^−1^. Then, the cells were diluted using saline to the final concentration of 10^5^ CFU mL^−1^. The obtained cell suspension was used for further experiments.

### 3.7. Antimicrobial Activity Test

The dark toxicity of RB-HA was examined in a comparison to different concentrations of RB. The 3 mL of suspensions containing cells 10^4^ CFU mL^−1^ with RB in concentrations of 0.1, 0.01, and 0.001 mg/mL and RB-HA in a concentration of 0.16 mg/mL were prepared. Then, the solutions were kept in a shaker at 100 rpm and in the dark for 10 and 20 min for *S. aureus* and *E. coli*, respectively. Then, 0.1 mL of the solutions were cultured on BH agar plates overnight at 37 ± 1 °C in the dark. The bacterial colony-forming units (CFU) were counted using a Scan 500 colony counter (Interscience, Saint-Nom-la Bretèche, France).

An antimicrobial activity test of the RB-HA conjugate was carried out in glass vials with lids for 80 days. The RB-HA (50 mg) was dissolved in saline (2.7 mL), and this volume was monitored during the test. The suspensions of the cells 10^5^ CFU mL^−1^ (0.3 mL) were added to the RB-HA solution before each experiment. Thus, the study was conducted under the same conditions at a cell concentration of 10^4^ CFU mL^−1^ in 3 mL of the mixture. During exposure to light or ultrasound for certain periods of time, 0.1 mL was taken from the samples and cultured on BH agar plates overnight at 37 ± 1 °C in the dark, and then the CFUs were counted. Between the experiments, the bottles with RB-HA were kept in the dark in an incubator at 25 °C.

Light irradiation was carried out using a 18 W LED lamp (ORSAM, model L18W/765, cool daylight, Munich, Germany), under shaking at 100 rpm. The lamp, which emitted light between 400 and 800 nm with a fluence rate of 7.1 mW cm^−2^ and light intensity of 35 klux, was used for all experiments. Light intensity was measured using an LX-102 light meter (Lutron, Taipei, Taiwan). The samples were placed in front of a lamp at a distance of 20 cm, and doses were 4 J cm^−2^ and 8 J cm^−2^ for *S. aureus* and *E. coli*, respectively.

Ultrasound exposure was carried out in an ultrasonic bath VUO3H (SMEG Instruments, Guastalla, Italy) at a frequency of 38 kHz. The sampling time was similar to that in the light irradiation experiment.

### 3.8. Statistical Analysis

The results obtained from at least three independent experiments carried out in duplicates were analyzed through single-factor ANOVA analyses. The differences between the results were considered significant when the *p*-value was less than 0.05. Quantitative results are presented as the mean ± standard error.

## 4. Conclusions

This paper presents a comprehensive analysis of the RB-HA conjugate we synthesized, providing insight into its composition, structure, singlet oxygen generation, photostability, and photo- and sonodynamic activity. The RB-HA conjugate demonstrated the ability to generate ROS upon exposure to LED light, indicating the potential for applications of the conjugate as a PS in photodynamic therapy (PDT). The singlet oxygen quantum yield (ΔΦ) of RB-HA under illumination with an LED lamp was close to the parameters of free RB. The RB-HA conjugate exhibited higher photostability and a slower photodegradation rate compared to free RB. The process of the photodegradation of RB-HA was three times slower than that of free RB. RB-HA demonstrated antibacterial activity against Gram-positive (*S. aureus*) and Gram-negative (*E. coli*) bacteria under visible light illumination and ultrasonication. Bacteria were either totally killed or strongly inhibited. The gradual release of RB from the conjugate ensured a constant and sufficient bactericidal concentration of PS in the medium over a long period (at least 80 days), wherein the conjugate did not exhibit dark toxicity towards bacterial cells. Thus, the RB-HA conjugate shows good prospects for use as an antibacterial agent with efficient singlet oxygen generation, improved photostability, and sustained antibacterial activity, making it a potential candidate for use in antimicrobial photodynamic and sonodynamic therapy in dental and other diseases.

## Data Availability

The data are available in the current publication.
